# Multilocus Sequence Types and Antimicrobial Resistance of *Campylobacter jejuni* and *C. coli* Isolates of Human Patients From Beijing, China, 2017–2018

**DOI:** 10.3389/fmicb.2020.554784

**Published:** 2020-10-19

**Authors:** Penghang Zhang, Xiaoai Zhang, Yuzhu Liu, Jinru Jiang, Zhangqi Shen, Qian Chen, Xiaochen Ma

**Affiliations:** ^1^Beijing Center for Disease Prevention and Control, Institute for Nutrition and Food Hygiene, Beijing, China; ^2^Beijing Centers for Disease Preventive Medical Research, Beijing, China; ^3^Beijing Advanced Innovation Center for Food Nutrition and Human Health, College of Veterinary Medicine, China Agricultural University, Beijing, China

**Keywords:** *Campylobacter* spp., human, MLST, antimicrobial susceptibility, China

## Abstract

*Campylobacter* species are zoonotic pathogens and the leading cause of bacterial enteritis worldwide. With the increase of antimicrobial resistance to fluoroquinolones and macrolides, they have been identified by the World Health Organization (WHO) as high-priority antimicrobial-resistant pathogens. There is currently little known about the prevalence and antimicrobial resistance characteristics of *Campylobacter* species in Beijing. In this study, we performed a 2-year surveillance of *Campylobacter* in Beijing, China. We used multilocus sequence typing (MLST) and antimicrobial susceptibility testing to analyze 236 *Campylobacter* isolates recovered from 230 clinical infectious cases in Beijing between 2017 and 2018. The *Campylobacter* isolation rate in diarrhea patients was 7.81%, with higher isolation rates in male patients than female patients and in autumn compared with other seasons. We identified 125 sequence types (STs) of 23 cloning complexes (CCs) among the 236 isolates, including four new alleles and 19 new STs. The most commonly isolated STs of *Campylobacter jejuni* were ST-22 and ST-760 (4.50%), and the most commonly isolated ST of *Campylobacter coli* was ST-9227 (16.67%). We also compared our isolates with clinical *Campylobacter* isolates from other countries in Asia, CC-353 of *Campylobacter coli* was found in eight countries, CC-1034 and CC-1287 of *Campylobacter coli* were found only in China. All *C. jejuni* isolates were resistant to at least one antimicrobial. *C. jejuni* showed the highest rate of resistance toward ciprofloxacin (94.50%), followed by tetracycline (93.50%), and nalidixic acid (92.00%), while *C. coli* showed highest resistance toward ciprofloxacin (94.44%) and tetracycline (94.44%) followed by nalidixic acid (88.89%). The most commonly observed MDR combination of *C. jejuni* were quinolone, phenicol and tetracycline (11.50%), while the most commonly observed MDR combination of *C. coli* were macrolide, quinolone, phenicol, tetracycline and lincosamide (30.56%). Surveillance of molecular characterization will provide important information for prevention of *Campylobacter* infection. This study enhances insight into *Campylobacter* infections in diarrheal patients, with relevance for treatment regimens in Beijing.

## Introduction

*Campylobacter* species are responsible for the greatest number of bacterial-mediated diarrhea outbreaks in the world (World Health Organization, 2013), with the thermophilic *Campylobacter jejuni* and *Campylobacter coli* being the most common pathogens. The European Food Safety Agency (EFSA) reports that *C. jejuni* has caused the most cases of bacterial foodborne diseases in the European Union for many years (European Food Safety Agency and European centre for Disease Prevention and Control, 2015). According to statistics on the number of foodborne pathogenic microorganisms in the United States by the United States Centers for Disease Control and Prevention (USA-CDC) in 2016, the number of cases caused by *C. jej*uni ranked highest (Centers for Disease Control and Prevention [CDC], 2017). In addition, *Campylobacter* infection can lead to extraintestinal complications and campylobacteriosis such as Guillain Barré syndrome, reactive arthritis, and irritable bowel syndrome (Nachamkin et al., 1998; Zia et al., 2003; Connor, 2005). An outbreak of *C. jejuni* resulted in 32 Guillain Barré syndrome patients in Shuangyang District, Changchun City, Jilin Province, China, during 2007 (Zhang et al., 2010b).

*Campylobacter* can colonize poultry, swine, and ruminants (Gallay et al., 2007; Elhadidy et al., 2018); therefore, people are easily infected when handling and consuming raw and semi-raw contaminated foods, as well as contaminated water (Klein-Jöbstl et al., 2016; Kovanen et al., 2016). Most *Campylobacter* infections are mild, self-limiting, and resolve within a few days. However, effective antimicrobial treatment is essential for people with severe or prolonged *Campylobacter* infections, and the elderly, young, or immunocompromised patients (Reddy and Zishiri, 2017). Clinical treatment of gastroenteritis caused by *Campylobacter* is usually performed with macrolides and fluoroquinolones; however, occasionally, severe systemic infection requires the use of aminoglycoside antimicrobials, such as gentamicin (Blaser and Engberg, 2008; Pernica et al., 2016). *Campylobacter* isolates from both developed and developing countries show resistance to several antimicrobials, including aminoglycosides, fluoroquinolones, macrolides, and tetracyclines (Padungton and Kaneene, 2003; Ruiz-Palacios, 2007; Ge et al., 2013; Abdi-Hachesoo et al., 2014; Shobo et al., 2016; Reddy and Zishiri, 2017), which led the World Health Organization in 2017 to list *Campylobacter* spp. as one of the six high-priority antimicrobial-resistant pathogens (World Health Organization, 2017). Therefore, identifying the molecular characteristics of resistant isolates and estimating their frequency in different populations is important for disease prevention aimed at controlling the emergence of resistant isolates and detecting resistant infections rapidly.

Molecular typing of bacteria plays an important role in the epidemiological investigation of pathogen transmission pathways by identifying outbreaks and diseases (O’Mahony et al., 2011). However, only a few studies have investigated the isolation rate and molecular characterization of clinical *Campylobacter* spp. in China (Zhang et al., 2010a, 2014; Wei et al., 2015; Pan et al., 2016; Ju et al., 2018). To understand the genotypes and antimicrobial resistance of clinical *Campylobacter* isolates in Beijing, we combined antimicrobial susceptibility tests and molecular subtyping analysis with epidemiological information.

## Materials and Methods

### Study Population and *Campylobacter* Isolation

From 2017 to 2018, *Campylobacter* isolates were obtained from 2,945 patients with acute diarrhea in 14 hospitals in pilot areas (Xicheng, Fengtai, Shunyi, Fangshan, Yanqing, and Mentougou Districts) of a *Campylobacter* surveillance program in Beijing. Patients were defined as having ≥3 watery, loose, mucosal, or bloody stools over a 24-h period. Five milligrams of fresh stool sample was collected from each of the diarrheal patients. The samples were maintained in Cary-Blair medium (Oxoid CM0935), at 4°C and transported to the laboratory for bacterial isolation within 24 h. A *Campylobacter* isolation kit incorporating a membrane filter method (ZC-CAMPY-002, Qingdao Sinova Biotechnology Co., Ltd., Qingdao, China) was used to isolate *Campylobacter*. Briefly, 1 mL of fecal specimen suspension was transferred to 4 mL of enrichment medium provided in the kit. The main component of the enrichment medium was a modified Preston broth. The enrichment medium was then cultured at 42°C for 48 h in a microaerobic atmosphere (5% O_2_, 10% CO_2_, and 85% N_2_). Approximately 300 μL of enrichment medium was spotted on the membrane filter (Type: 0.45 μm) surface of the kit and spread onto Karmali and Columbia agar plates. Five (if less than 5, pick all) or more colonies resembling *Campylobacter* were picked after 48 h of incubation at 42°C in a microaerobic atmosphere. All isolates were firstly identified using matrix-assisted laser desorption/ionization time of flight (MALDI-TOF) mass spectrometry (Bruker, Leipzig, Germany) and further checked by PCR, according to a previously described method (Wang et al., 2002).

### Epidemiological Data

Demographic and epidemiological data were managed using WPS Office (Kingsoft, China). All patients were residents of Beijing. Patients were divided according to age into child patients (≤5 years old), adolescent patients (6–17 years old), adult patients (18–64 years old), and elderly patients (≥65 years old). Seasons were classified according to the onset date of symptoms: spring (March, April, May), summer (June, July, August), autumn (September, October, November), and winter (December, January, February).

### Multilocus Sequence Typing

MLST was performed by sequencing seven housekeeping loci (aspA, glnA, gltA, glyA, pgm, tkt, and uncA) according to a previously described method (Dingle et al., 2001). Briefly, genomic DNA was extracted using a commercial kit (QIAamp DNA mini kit, Germany). Amplification reactions were performed in a 20 μL mixture containing 10 μL of 2 × PCR master (Sangon Biotec., Shanghai, China), 0.5 μL of forward and reverse primer, 1 μL of DNA template, and 7.5 μL of pure water. PCR was performed as 1 cycle of 5 min at 94°C; 35 cycles of 1 min at 94°C, 2 min at 50°C, and 1 min at 72°C; and a final extension of 5 min at 72°C. After amplification, 2 μL of the PCR product was subjected to electrophoresis through a 1.5% agarose gel. The PCR products were then purified and sequenced (3730xl DNA Analyzer, Applied Biosystems, United States). The alleles of each gene were determined from a comparison of seven alleles in the MLST database^1^. For each isolate, the number of alleles was assigned and sequence types (STs) and cloning complexes (CCs) were determined. New alleles and STs were submitted to the PubMLST database. A minimum spanning tree (MST) and dendrogram of MLST data was created using BioNumerics v.7.6 (bioMérieux, Marcy-l’Étoile, France).

### Antimicrobial Susceptibility Testing

The minimum inhibitory concentration (MIC) of all *C. jejuni* isolates was determined according to the agar dilution method recommended by CLSI using a commercial kit (Zhongchuang Biotechnology Ltd. Corp., Qingdao, China). Six classes of antimicribials were chosen for this study: macrolides, fluoroquinolones, aminoglycosides, chloramphenicols, tetracyclines, and lincosamides. The breakpoints for resistance used in this study were based on standards used in the National Antimicrobial Resistance Monitoring System (NARMS, Page last reviewed: March 15, 2019). The following MIC values were determined for C. jejuni: erythromycin (≥8 μg mL^–1^), azithromycin (≥0.5 μg mL^–1^), nalidixic acid (≥32 μg mL^–1^), ciprofloxacin (≥1 μg mL^–1^), gentamicin (≥4 μg mL^–1^), streptomycin (≥16 μg mL^–1^), chloramphenicol (≥32 μg mL^–1^), florfenicol (≥8 μg mL^–1^), tetracycline (≥2 μg mL^–1^), telithromycin (≥8 μg mL^–1^), and clindamycin (≥1 μg mL^–1^). MIC values for C. coli were as follows: erythromycin (≥16 μg mL^–1^), azithromycin (≥1 μg mL^–1^), nalidixic acid (≥32 μg mL^–1^), ciprofloxacin (≥1 μg mL^–1^), gentamicin (≥4 μg mL^–1^), streptomycin (≥16 μg mL^–1^), chloramphenicol (≥32 μg mL^–1^), florfenicol (≥8 μg mL^–1^), tetracycline (≥4 μg mL^–1^), telithromycin (≥8 μg mL^–1^), and clindamycin (≥2 μg mL^–1^). Multi-drug resistance (MDR) was defined as resistance to three or more classes of antimicrobials in this study. *C. jejuni* ATCC 33560 was used as a control.

### Statistical Analysis

SPSS software, version 20.0 (SPSS, Inc.) was used to analyze any significant statistical differences in the results. Differences in the frequencies of *Campylobacter* isolation rate across STs, CCs, and MDR isolates, and other variables, including epidemiological data, were examined using χ^2^ and Fisher’s exact tests for dichotomous variables; *p* < 0.05 was considered significant.

## Results

### Description of *Campylobacter* Cases Identified and Prevalence of *Campylobacter* spp. in Beijing

We collected 2,945 stool samples from individual diarrhea patients in 14 hospitals in Beijing during 2017 and 2018; 236 *Campylobacter* isolates were recovered, comprising 200 isolates of *C. jejuni* (84.75%, 200/236) and 36 isolates of *C. coli* (15.25%, 36/236). There were 230 positive cases of *Campylobacter* infection, including five cases with co-infections. Among these, three cases were co-infected with *C. jejuni* and *C. coli*, one case was co-infected with two *C. jejuni* isolates, and one case was co-infected with two *C. jejuni* isolates and one *C. coli* isolate.

The isolation rate of *Campylobacter* in diarrhea patients was 7.81% (230/2945). Of these patients, 148 were male (64.35%) while 82 were female (35.65%). Patients ranged in age from 3 to 91 years old. *Campylobacter* isolation rate was higher in autumn than in other seasons (spring, χ^2^ = 3.4909, *P* < 0.0682; summer, χ^2^ = 6.8528, *P* < 0.0105; winter, χ^2^ = 6.7567, *P* < 0.0130), and higher in male than in female (χ^2^ = 8.6054, *P* < 0.0037) (Table 1). No statistically significant difference was found in the *Campylobacter* isolation rate between the child and other age groups (adolescent, χ^2^ = 0.9406, *P* < 0.3321; adult, χ^2^ = 0.3216, *P* < 0.5707; elderly, χ^2^ = 0.0012, *P* < 0.9719).

**TABLE 1:** Description of the patients (230, total number of studied 2,945) included in this study.

Epidemiological data	No. of cases (%)	No. of patients (percentage of isolation rate)	χ^2^	P
**Sex**			8.6054	0.0037
Male	148 (64.35%)	148/1623 (9.12%)		
Female	82 (35.65%)	82/1322 (6.20%)		
**Age group**			6.1753	0.0983
Child	1 (0.43%)	1/29 (3.45%)		
Adolescent	12 (5.22%)	12/102 (11.76%)		
Adult	196 (85.22%)	196/2429 (8.07%)		
Elderly	21 (9.13%)	21/385 (5.45%)		
**Season**			11.0292	0.0116
Spring	51 (22.17%)	51/682 (7.48%)		
Summer	86 (37.39%)	86/1231 (6.99%)		
Autumn	82 (35.65%)	82/799 (10.26%)		
Winter	11 (4.78%)	11/233 (4.72%)		

### Multilocus Sequence Typing

**FIGURE 1:**
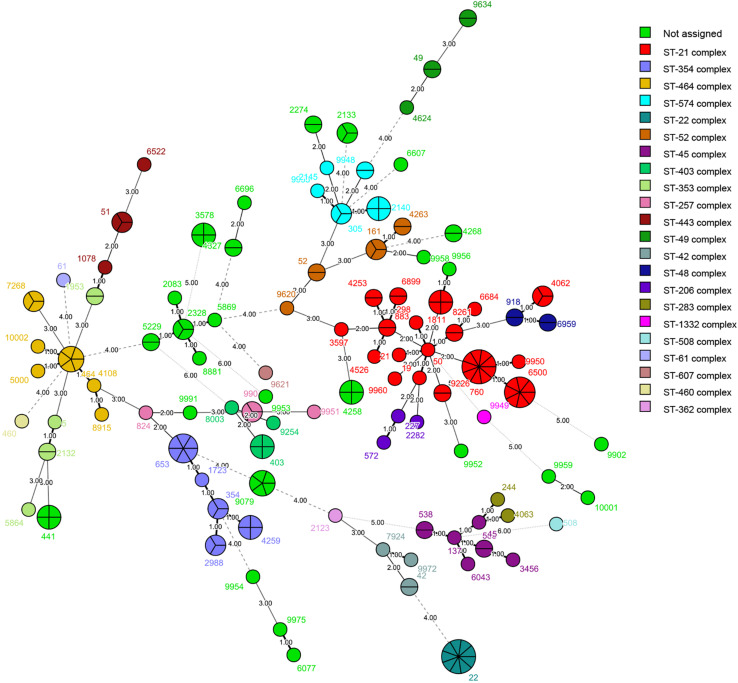
Phylogenetic analysis. Minimum spanning tree (MST) of MLST data of 200 *C. jejuni* human isolates collected in 2017 and 2018 in Beijing, China. Each color represents one clonal complex (CC). Isolates are represented by circles, and the size of the circle is proportional to the number of isolates. Branches and numbers represent allelic differences between isolates.

**FIGURE 2:**
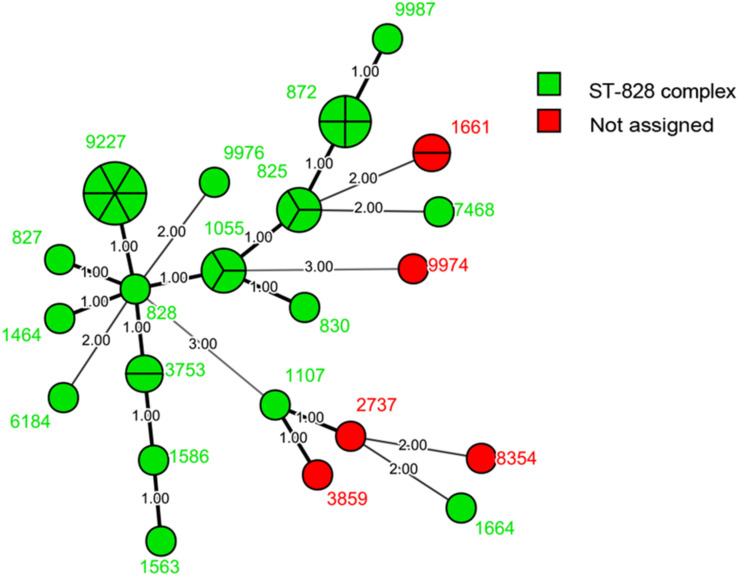
Phylogenetic analysis. Minimum spanning tree (MST) of MLST data of 36 *C. coli* human isolates collected in 2017 and 2018 in Beijing, China. Green represents the ST-828 complex and red represents isolates that do not belong to any known clonal complex. Isolates are represented by circles, and the size of the circle is proportional to the number of isolates. Branches and numbers represent allelic differences between isolates.

We identified 125 STs of 23 CCs among the 236 isolates, including four new alleles and 19 new STs. The new STs are shown in Supplementary Table S1. Thirty STs from 53 isolates did not belong to any known clonal complex. Among the 200 isolates of *C. jejuni*, we recovered 103 STs. The most commonly isolated STs were ST-22 and ST-760 (9/200, 4.50%), followed by ST-6500 (7/200, 3.50%), and ST-653 (6/200, 3.00%); 59 STs had only one isolate (Figure 1). The most commonly isolated CC was CC-21 (42/200, 21.00%). Among 36 isolates of *C. coli*, we recovered 22 STs. The most commonly isolated was ST-9227 (6/36, 16.67%). CC-828 was the only CC found in *C. coli* (30/36, 83.33%), and six isolates did not belong to any known CC (Figure 2).

### Comparison of Isolates From This Study With Those From Other Districts in China

**FIGURE 3:**
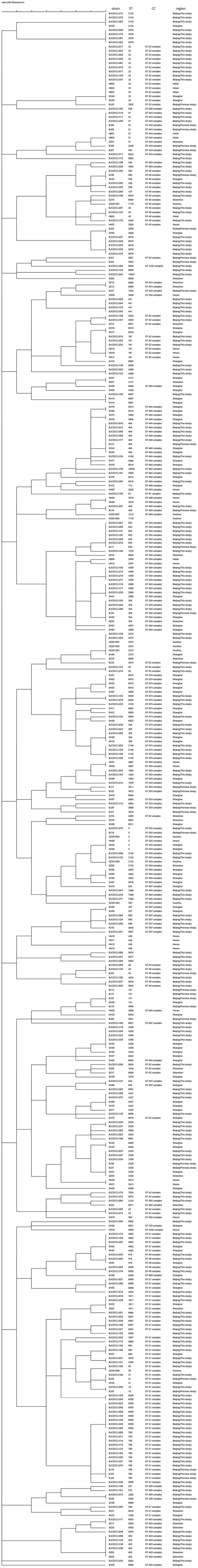
Dendrogram of MLST data of C. *jejuni* human isolates collected from this study and other district of China, Showed the number of differences in MLST alleles. The similarity coefficient were calculated using the categorical (difference) and complete linkage for the cluster analysis.

**FIGURE 4:**
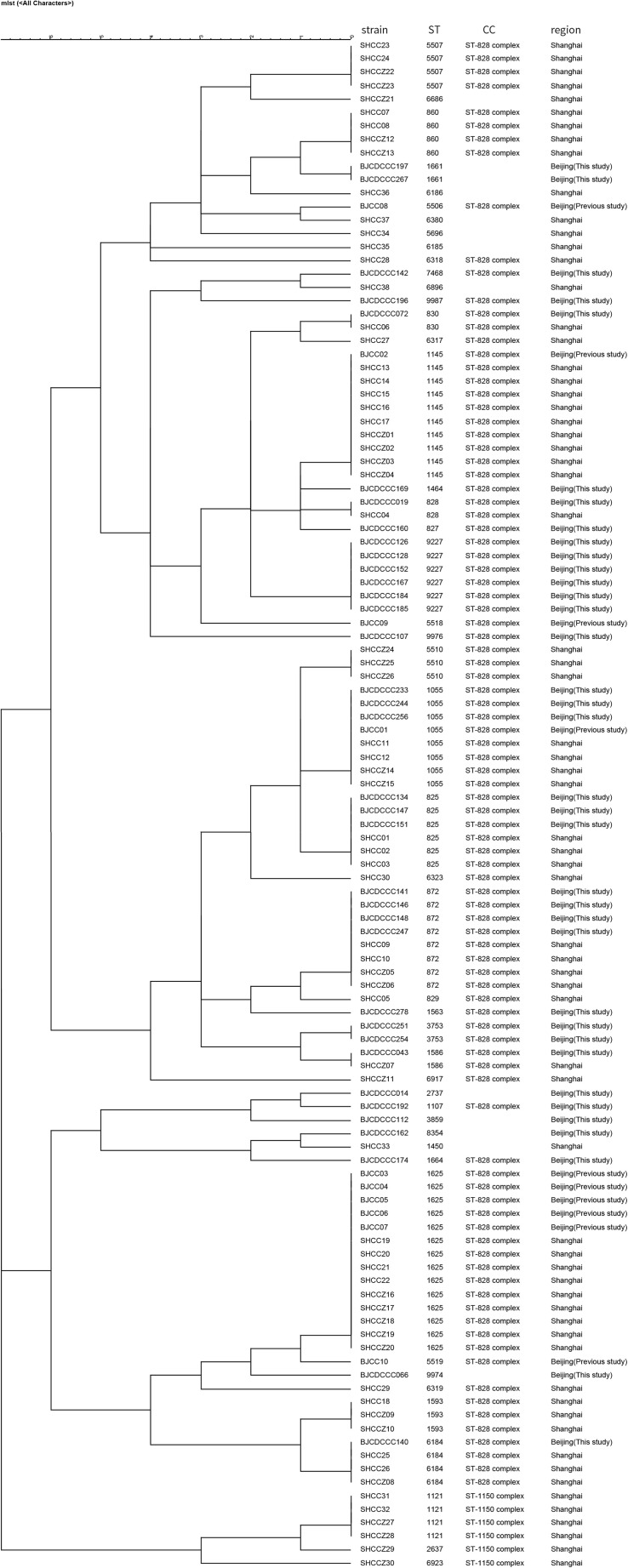
Dendrogram of MLST data of *C. coli* human isolates collected from this study and other district of China, showed the number of differences in MLST alleles. The similarity coefficient were calculated using the categorical (difference) and complete linkage for the cluster analysis.

We compared the distribution of *Campylobacter* isolates from diarrhea patients from Beijing with those from patients from other districts in China using MLST allelic profiles available from previous reports (Zhang et al., 2010a, 2014, 2015; Wei et al., 2015; Pan et al., 2016; Ju et al., 2018). We constructed dendrograms for *C. jejuni* (Figure 3) and *C. coli* (Figure 4) isolates. In total, 187 STs were recovered from 376 isolates of *C. jejuni*. There were no obvious regional characteristics for these STs: ST-5 (*n* = 6) was recovered from four districts while ST-22 (*n* = 14, most common), ST-51 (*n* = 8), ST-2328 (*n* = 7), ST-354 (*n* = 6), ST-2274 (*n* = 6), ST-1811 (*n* = 4), and ST-2132 (*n* = 4) were recovered from three districts of China. A total of 47 STs were recovered from 114 isolates of *C. coli*. Among these, nine STs were found in both Beijing and Shanghai City. CC-21 (57/376, 15.16%) and CC-828 (95/114, 83.33%) were the most commonly isolated CCs for *C. jejuni* and *C. coli*, respectively.

### Comparison of Isolates With Those From Other Countries in Asia

**FIGURE 5:**
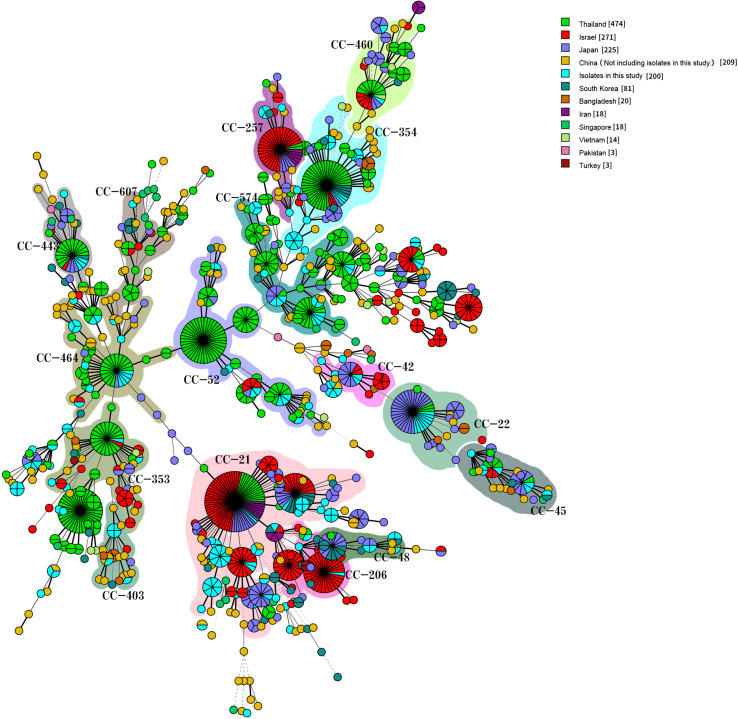
Genetic relationships among the 200 clinical isolates *C. jejuni* in this study and 1,336 isolates *C. jejuni* from Asia in the pubmlst database (Data taken time: January 18, 2019). A minimum spanning tree was reconstructed based on CCs from this study and the MLST database. The size of circles is proportional to the number of isolates, and the sources of the isolates are colored as indicated. Shadow zones in different colors represent different clonal complexes.

**FIGURE 6:**
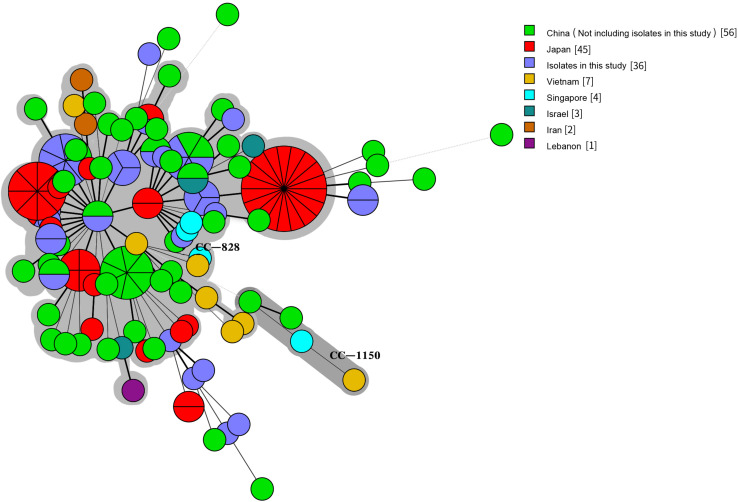
Genetic relationships among the 36 clinical isolates *C. coli* in this study and 118 isolates *C. coli* from Asia in the pubmlst database (Data taken time: January 18, 2019). A minimum spanning tree was reconstructed based on CCs from this study and the MLST database. The size of circles is proportional to the number of isolates, and the sources of isolates are colored as indicated. Shadow zones in different color represent different clonal complexes.

We compared our isolates with clinical *Campylobacter* isolates from other countries in Asia by selecting 1,454 *Campylobacter* isolates from patients with diarrhea from the PubMLST database (see text footnote 1) on January 18, 2019: 1336 *C. jejuni* and 118 *C. coli* isolates. The 1,536 human *C. jejuni* isolates were divided into 631 STs and 30 CCs, with 22 of these CCs found in this study. The most prevalent CCs were CC-21 (259/1536, 16.86%), CC-52 (105/1536, 6.84%), CC-353 (104/1536, 6.77%), CC-354 (102/1536, 6.64%), CC-574 (100/1536, 6.51%), CC-464 (67/1536, 4.36%), CC-257 (53/1536, 3.45%), and CC-22 (53/1536, 3.45%). CC-353 was found in eight countries, CC-21, CC-443 (41/1536, 2.67%), CC-45 (37/1536, 2.41%), and CC-574 were found in seven countries, and CC-206 (45/1536, 2.93%) and CC-257 were found in six countries. CC-1034 and CC-1287 were found only in China, CC-692 and CC-177 were found only in Thailand, and CC-41 and CC-446 were found only in Bangladesh and Israel, respectively (Figure 5). The 154 human *C. coli* isolates were divided into 95 STs and 2 CCs. One hundred and thirty-one isolates belonged to CC-828, 3 isolates from different countries belonged to CC-1150, and the remaining isolates did not belong to any known CC (Figure 6).

### Antibacterial Susceptibility Testing

The MICs of the quality control isolate (*C. jejuni* ATCC 33560) were within the reference quality control range. No *C. jejuni* isolate was susceptible to all 11 antimicrobials tested; however, 179 *C. jejuni* isolates (89.50%) were resistant to three or more antimicrobials and two isolates were susceptible to only one antimicrobial (erythromycin and gentamicin, respectively). *C. jejuni* showed highest resistance to ciprofloxacin (94.50%), followed by tetracycline (93.50%), and nalidixic acid (92.00%) (Table 2). The most prevalent resistance pattern was a combination of nalidixic acid, ciprofloxacin, and tetracycline (33.33%) (Supplementary Figure S1). *C. coli* showed highest resistance to ciprofloxacin (94.44%) and tetracycline (94.44%), followed by nalidixic acid (88.89%). Thirty-five *C. coli* isolates (97.22%) were resistant to three or more antimicrobials, three isolates were resistant to all antimicrobials tested except chloramphenicol, and nine isolates were resistant to all antimicrobials except chloramphenicol and flofenicol; this was the most common resistance pattern. No *C. coli* isolates were resistant to chloramphenicol and one isolate was susceptible to all 11 antimicrobials (Supplementary Figure S2).

**TABLE 2:** Resistance rates (percentages) and minimum inhibitory concentrations (MICs) of human *Campylobacter* spp. isolates in Beijng.

*Campylobacter* spp.	Antibiotic	Resistance rate (%)	MIC (μg/mL)		
			
			Range	MIC_50_	MIC_90_
*C. jejuni* (*n* = 200)	Erythromycin	9.00	<0.5–>64	<0.5	4
	Azithromycin	13.00	<0.5–>64	<0.5	1
	Nalidixic acid	92.00	<0.5–>64	32	>64
	Ciprofloxacin	94.50	<0.5–>64	32	>64
	Gentamicin	13.00	<0.5–>64	<0.5	64
	Streptomycin	9.50	<0.5–>64	1	4
	Chloramphenicol	12.00	<0.5–>64	8	32
	Florfenicol	35.00	<0.5–>64	4	16
	Tetracycline	93.50	<0.5–>64	>64	>64
	Telithromycin	12.00	<0.25–>32	1	8
	Clindamycin	33.50	<0.25–>32	0.5	1
*C. coli* (*n* = 36)	Erythromycin	44.44	<0.5–>64	4	>64
	Azithromycin	44.44	<0.5–>64	<0.5	>64
	Nalidixic acid	88.89	4–>64	>64	>64
	Ciprofloxacin	94.44	<0.5–>64	16	>64
	Gentamicin	50.00	<0.5–>64	2	>64
	Streptomycin	72.22	<0.5–>64	64	>64
	Chloramphenicol	0.00	1–16	8	16
	Florfenicol	16.67	<0.5–16	4	8
	Tetracycline	94.44	1–>64	>64	>64
	Telithromycin	63.89	<0.25–>32	8	>32
	Clindamycin	44.44	<0.25–>32	1	>32

The MIC_50_ and MIC_90_ values for *Campylobacter* are shown in Table 2. Statistical differences for antibacterial susceptibility between different antimicrobials are shown in Supplementary Table S2. Statistically, *C. jejuni* exhibited lower resistance rates to erythromycin (χ^2^ = 31.0821, *P* < 0.0001), azithromycin (χ^2^ = 20.6197, *P* < 0.0001), gentamicin (χ^2^ = 27.5356, *P* < 0.0001), streptomycin (χ^2^ = 77.7753, *P* < 0.0001), and telithromycin (χ^2^ = 51.5031, *P* < 0.0001) than *C. coli*, while the *C. coli* resistance rate to florfenicol (χ^2^ = 4.6967, *P* = 0.0332) was lower than that of *C. jejuni*. In addition, among 8–10 antimicrobial-resistant isolates, the isolation rate of *C. coli* was higher than that of *C. jejuni* (χ^2^ = 26.2338, *P* < 0.0001).

For multi-drug resistance (MDR), 110 *C. jejuni* isolates (55.00%) were resistant to three or more classes of antimicrobials. Among these, 23 isolates (11.50%) were resistant to quinolone, phenicol and tetracycline, simultaneously 21 isolates (10.50%) were resistant to quinolone, phenicol, tetracycline and lincosamide. Seven isolates (3.50%) were resistant to all six classes of antimicrobials. We also recovered 27 MDR isolates of *C. coli* (75.00%), of which 11 isolates (30.56%) were resistant to macrolide, quinolone, phenicol, tetracycline, and lincosamide. Three isolates (8.33%) were resistant to all six classes of antimicrobials.

We detected 81 STs representing 19 CCs from 137 MDR isolates. Among these, ST-872, ST-1055, ST-2140, ST-9959, and ST-9976 were resistant to 10 antimicrobials (ST-872, ST-1055, and ST-9976 were sensitive to chloramphenicol. ST-2140 and ST-9959 were sensitive to gentamicin and erythromycin, respectively). Moreover, all ST-9226 isolates (*n* = 3) were resistant to 8–9 antimicrobials (sensitive to streptomycin, chloramphenicol, and florfenicol). All ST-1811 isolates (*n* = 2) were resistant to six antimicrobials (both isolates were resistant to nalidixic acid, ciprofloxacin chloramphenicol, florfenicol, and tetracycline, however one isolate was resistant to clindamycin and the other was resistant to gentamicin). Eleven isolates of CC-828 (11/30, 36.67%) were resistant to 8–10 antimicrobials, and three isolates (ST-403) of CC-403 (3/6, 50.00%) were resistant to eight antimicrobials. This suggested that the above STs and CCs are related to MDR of *Campylobacter* spp.

## Discussion

*Campylobacter* is one of the major causes of gastroenteritis in the world. The transmission chain of *Campylobacter* spp. is not completely known but chickens are considered to be the major reservoir for transmission to humans. Additional sources of infection are likely to include red meat, unpasteurized milk, and contaminated water (Mylius et al., 2007; Pernica et al., 2016). The present study provides more complete and updated information about the prevalence, antimicrobial resistance, and genetic diversity of *Campylobacter* isolates during 2017 and 2018 in Beijing. *Campylobacter* infections can be fatal among very young children (World Health Organization, 2020). Several studies of *Campylobacter* prevalence in children have been conducted previously. For instance, Zhu et al. (2016) reported that 2.9% of children with diarrhea were infected by *Campylobacter* in Wuhan (southwest China). Ju et al. (2018) reported that 4.0% of child patients with diarrhea were infected by *Campylobacter* in Shenzhen (southern China). Our study revealed a *Campylobacter* prevalence of 3.45% (1/29) in child patients with diarrhea, which was not statistically significantly different from other age groups. Moreover, Wang et al. (2015) reported no *Campylobacter* infections in child patients with diarrhea in Beijing. The discrepancy in prevalence may be caused by the different choice of surveillance hospitals. Only one of the 19 hospitals selected in this study is a children’s hospital. Our results indicated that the isolation rate of *Campylobacter* is higher in autumn than in other seasons. We suggest that attention should be paid to food hygiene in this season to reduce the risk of foodborne diseases.

In this study, we also highlighted the higher genetic diversity of *Campylobacter* isolates circulating in Beijing. We identified a total of 125 STs including 19 new STs, with the new STs reaching ST-10002 in this study. Data from an earlier study involving patient diarrheal isolates of *Campylobacter* collected in China revealed that the most commonly isolated ST in Hebei province is ST-22, consistent with our study. However, the most commonly isolated STs in Guizhou province, Henan province, Shanghai city, and Shenzhen city are ST-2274, ST-436, ST-6915, and ST-403, respectively (Zhang et al., 2010a; Wei et al., 2015; Pan et al., 2016; Ju et al., 2018). This difference in STs may be due to the low number of isolates. The most commonly isolated CCs in this study were CC-21 (42/200, 21.00%) and CC-828 (30/36, 83.33%) from *C. jejuni* and *C. coli*, respectively. Many studies have shown different major CCs of *Campylobacter* in different countries and regions, but CC-21, CC-45, CC-48, and CC-353 are the major CCs containing the largest number of isolates in many studies (O’Mahony et al., 2011; Klein-Jöbstl et al., 2016; Kovanen et al., 2016; Pernica et al., 2016; Dunn et al., 2018; Rokney et al., 2018). Studies have also shown that CC-21 is the most commonly isolated CC of *C. jejuni* in China (Zhang et al., 2010a, 2014, 2015; Wei et al., 2015; Pan et al., 2016; Ju et al., 2018), including human and chicken isolates. MLST analyses of *Campylobacter* in Asian diarrhea patients show that CC-21 is the most commonly isolated CC of *C. jejuni* in China, Israel, Iran, Japan, and South Korea. CC-1150 of *C. coli* has been found in Vietnam, Singapore, and China. These data highlight the diversity of *Campylobacter* spp. in Beijing and confirm previous MLST studies with *C. jejuni* and *C. coli* isolates from humans in China.

ST-2274 of *C. jejuni* and ST-9227 of *C. coli* caused outbreaks of foodborne diseases resulting in seven and three patients with diarrhea in Beijing, respectively (Qu et al., 2019; Li et al., 2020). ST-2274 was found in Thailand and Guizhou province and Shanghai city of China. ST-9227 has not been reported in the PubMLST database in Asia except in Beijing. An outbreak of Guillain Barré syndrome caused by *C. jejuni* was previously reported in Changchun City, Jilin Province, China, and the strains isolated from patients were all ST-2993 (Zhang et al., 2010b). However, no other ST-2993 isolates in the PubMLST database were from other country of Asia.

Antimicrobial resistance of *Campylobacter* is receiving more attention. Infection with antimicrobial-resistant isolates is often associated with longer disease duration, higher risk of invasive disease, and higher healthcare costs (Lopes et al., 2019). The use of antimicrobials in animal agriculture and human medicine can affect the development of resistance in *Campylobacter*. It is believed that the unrestricted use of antimicrobials, especially in developing countries, has led to increased resistance to *Campylobacter* spp. (Gibreel and Taylor, 2006; Deng et al., 2015). In some countries, in contrast to therapeutic agents, antibacterial agents are still used as growth promoters (Osterlund et al., 2003; Gibreel and Taylor, 2006; Qin et al., 2011; Wang et al., 2014). Antimicrobial-resistant strains of *Campylobacter* are a zoonotic pathogens that can be transmitted to humans through contaminated food, water, or milk, seriously jeopardizing treatment (Smith et al., 1999; Piddock et al., 2000; Engberg et al., 2004; Smith and Fratamico, 2010; Schweitzer et al., 2011). Determination of the antimicrobial resistance level of *Campylobacter* isolates is crucial for the control and prevention of human infection, particularly for cases in which therapy is recommended. Macrolides and fluoroquinolones are the primary choice for treating campylobacteriosis (Chen et al., 2010). However, an increase in erythromycin-resistant *Campylobacter* in poultry has been observed in China recently (Zhang A. et al., 2016). In our study, 44.44% of *C. coli* were resistant to erythromycin. We also found that 75.00% of *C. coli* were MDR isolates and 11 isolates (30.56%) were resistant to macrolide, quinolone, phenicol, tetracycline, and lincosamide. Therefore, the clinical treatment of campylobacteriosis caused by *C. coli* should be carefully reconsidered. The overall resistance level of *C. jejuni* is not as high as that of *C. coli*, but it cannot be ignored. High resistance rates of *C. jejuni* to quinolone and tetracycline, with a high prevalence of MDR, has been reported in China previously (Zhang T. et al., 2016; Li et al., 2017). In our study, 55.00% of *C. jejuni* were MDR isolates, with quinolone, phenicols, and tetracycline the most commonly observed MDR combination (11.50%), followed by quinolone, phenicol, tetracycline and lincosamides (10.50%). A significant association between ST-464 and resistance to ciprofloxacin, nalidixic acid, and tetracycline (cip-nal-tet) has been reported (Cha et al., 2016). Five isolates of ST-464 were recovered in our study, all of which were resistant to cip-nal-tet. One study also claims that ST-403 has strong antimicrobial resistance (Ju et al., 2018), which was shown in our study. Some studies, such as in Michigan and Iran (Cha et al., 2016; Divsalar et al., 2019), reported low resistance rate of *C. jejuni* to florfenicol and clindamycin. However, Higher resistance rates of *C. jejuni* to these two antimicrobials have been reported in China. Pan et al. (2016) reported 21.8% of *C. jejuni* isolated from children with diarrhea were resistant to clindamycin. Ju et al. (2018) reported florfenicol resistance rate of *C. jejuni* close to 70%. A 17-year study of *C. jejuni* resistance in diarrhea patients of Beijing showed that the resistance rate of florfenicol and clindamycin is increasing (Zhou et al., 2016). Alarming was resistance of *C. jejuni* to florfenicol (35.00%) and clindamycin (33.50%) in our study. We found 17 ioslates resistant to florfenicol, clindamycin and chloramphenicol. At the same time, all 17 ioslates were resistant to cip-nal-tet, and belong to 17 different STs. The resistance pattern of these antimicrobials limits the choice of suitable antimicrobials for human *Campylobacter* disease. Thus, we suggest that clinicians should consider multi-drug treatment.

An isolate of *C. coli* (ST-825, CC-828) sensitive to all antimicrobials was isolated from an adult man who claimed to have eaten seafood at home (unpublished data). We are very curious about this result, because in addition to this isolate, other *C. coli* isolates were resistant to two or more classes of antimicrobials. Therefore, the detection of resistance genes in *C. coli* is the focus of our next research.

In China, a few studies have reported the prevalence of *Campylobacter* in food and animals (Zhang et al., 2015; Han et al., 2016; Zhu et al., 2017). However, descriptions of clinical *Campylobacter* are very limited. In this study, we described the characteristics of molecular typing and antimicrobial susceptibility profiles of clinical *Campylobacter* isolates in Beijing, the capital city of China. The clinical isolates analyzed in this study were recovered from a systematic surveillance program, which provided a unique opportunity to characterize *Campylobacter* spp. in Beijing.

## Conclusion

Based on our results, attention should be drawn to the prevalence of isolates with high antimicrobial resistance rates and genetic similarity in Beijing and other districts of China. Food manufacturers should continually assess levels of contamination in food production and processing to effectively decrease *Campylobacter* infection and dissemination of resistant isolates in humans. Importantly, this study provides detailed insight into the phylogeny and antimicrobial resistance profiles of *Campylobacter* spp. in Beijing. Further surveillance is necessary to detect emerging resistance patterns and to assess the impact of strategies designed to mitigate antimicrobial resistance.

## Data Availability Statement

All relevant data is contained within the article. All datasets generated for this study are included in the article/Supplementary Material, further inquiries can be directed to the corresponding authors.

## Ethics Statement

The protocol was approved by the ethics committee of Beijing Center for Disease Prevention and Control (Beijing CDC). Participants received information on the study’s purpose and of their right to keep information confidential. Written consent was obtained from each participant and children’s parents or their guardians.

## Author Contributions

JJ and XM were involved in the collection of isolates and collected the clinical data. YL, XZ, and PZ performed the molecular subtyping and antibiotic susceptibility tests. XZ, JJ, and PZ performed the data analysis. PZ, XZ, ZS, XM, and QC designed the study, drafted, and revised this manuscript. All authors contributed to the article and approved the submitted version.

## Conflict of Interest

The authors declare that the research was conducted in the absence of any commercial or financial relationships that could be construed as a potential conflict of interest.

## Footnotes

1 http://pubmlst.org/campylobacter/
