# Supplementary material for: Multilocus Sequence Types and Antimicrobial Resistance of Campylobacter jejuni and C. coli Isolates of Human Patients From Beijing, China, 2017–2018
Source: Front Microbiol. 2020 Oct 19;11:554784. doi: 10.3389/fmicb.2020.554784 (PMC7604515; doi:10.3389/fmicb.2020.554784)
Supplement: Supplementary file 1 [file Table_1.docx]

Supplementary Table S1. New STs and alleles of 23 isolates from Beijing

| Isolates | aspA | glnA | gltA | glyA | pgm | tkt | uncA | ST | Clonal complex | PubMLST database id |
| --- | --- | --- | --- | --- | --- | --- | --- | --- | --- | --- |
| BJCDCCJ115 | 2 | 1 | 42 | 3 | 2 | 3 | 5 | 9960 | ST-21 complex | 80317 |
| BJCDCCJ133 | 37 | 367 | 292 | 28 | 127 | 25 | 541 | 9959 |  | 80316 |
| BJCDCCJ136 | 14 | 21 | 2 | 10 | 127 | 289 | 6 | 9958 |  | 80315 |
| BJCDCCJ153 | 2 | 609 | 12 | 3 | 11 | 3 | 5 | 9956 |  | 80313 |
| BJCDCCJ154 | 9 | 53 | 2 | 10 | 13 | 3 | 3 | 9955 | ST-574 complex | 80312 |
| BJCDCCC196 | 33 | 39 | 30 | 140 | 113 | 44 | 17 | 9987 | ST-828 complex | 80357 |
| BJCDCCJ206 | 8 | 71 | 5 | 62 | 11 | 67 | 6 | 9954 |  | 80310 |
| BJCDCCJ224 | 8 | 61 | 80 | 144 | 220 | 772 | 533 | 9953 |  | 80309 |
| BJCDCCJ240 | 2 | 1 | 2 | 26 | 127 | 29 | 35 | 9952 |  | 80308 |
| BJCDCCJ241 | 1 | 2 | 10 | 62 | 4 | 5 | 6 | 9951 | ST-257 complex | 80307 |
| BJCDCCJ246 | 2 | 1 | 52 | 3 | 74 | 100 | 5 | 9950 | ST-21 complex | 80306 |
| BJCDCCJ264 | 2 | 1 | 4 | 28 | 470 | 25 | 58 | 9949 | ST-1332 complex | 80305 |
| BJCDCCJ279 | 9 | 731 | 5 | 10 | 11 | 3 | 3 | 9948 | ST-574 complex | 80304 |
| BJCDCCJ063 | 9 | 2 | 2 | 6 | 11 | 133 | 6 | 9991 |  | 80476 |
| BJCDCCJ222 | 1 | 2 | 42 | 4 | 1025 | 9 | 467 | 9972 | ST-42 complex | 80339 |
| BJCDCCC066 | 32 | 39 | 30 | 82 | **1029^#^** | 47 | 139 | 9974 |  | 80341 |
| BJCDCCJ006 | 7 | 71 | 5 | 62 | **1028** | 67 | 26 | 9975 |  | 80342 |
| BJCDCCJ243 | 37 | 367 | **632** | 28 | 127 | 29 | 541 | 10001 |  | 80577 |
| BJCDCCJ159 | 24 | 2 | 2 | 2 | 10 | 3 | **662** | 10002 | ST-464 complex | 80578 |

BJCDCCC196 and BJCDCCC066 are *C. coli*, the others are *C. jejuni*.

^#^New alleles are given in bold.
