# Supplementary material for: Multilocus Sequence Types and Antimicrobial Resistance of Campylobacter jejuni and C. coli Isolates of Human Patients From Beijing, China, 2017–2018
Source: Front Microbiol. 2020 Oct 19;11:554784. doi: 10.3389/fmicb.2020.554784 (PMC7604515; doi:10.3389/fmicb.2020.554784)
Supplement: Supplementary file 2 [file Table_2.docx]

Supplementary Table S2. Statistical difference for the antibacterial susceptibility

between different antibiotics

| *C. jejuni* (n=200) | **χ^2^** | P | *C. coli* (n=36) | **χ^2^** | P |
| --- | --- | --- | --- | --- | --- |
| Erythromycin vs. Azithromycin | 1.6343 | 0.2632 | Erythromycin vs. Azithromycin | 0.0000 | 1.0000 |
| Erythromycin vs. Nalidixic acid | 275.5876 | ＜0.0001 | Erythromycin vs. Nalidixic acid | 16.0000 | 0.0001 |
| Erythromycin vs. Ciprofloxacin | 292.7686 | ＜0.0001 | Erythromycin vs. Ciprofloxacin | 21.2073 | ＜0.0001 |
| Erythromycin vs. Gentamicin | 1.6343 | 0.2632 | Erythromycin vs. Gentamicin | 0.2229 | 0.8136 |
| Erythromycin vs. Streptomycin | 0.0298 | 1.0000 | Erythromycin vs. Streptomycin | 5.7143 | 0.0307 |
| Erythromycin vs. Chloramphenicol | 0.9577 | 0.4151 | Erythromycin vs. Chloramphenicol | 20.5714 | ＜0.0001 |
| Erythromycin vs. Florfenicol | 39.3939 | ＜0.0001 | Erythromycin vs. Florfenicol | 6.5455 | 0.0202 |
| Erythromycin vs. Tetracycline | 285.7886 | ＜0.0001 | Erythromycin vs. Tetracycline | 21.2073 | ＜0.0001 |
| Erythromycin vs. Telithromycin | 0.9577 | 0.4151 | Erythromycin vs. Telithromycin | 2.7413 | 0.1554 |
| Erythromycin vs. Clindamycin | 35.8693 | ＜0.0001 | Erythromycin vs. Clindamycin | 0.0000 | 1.0000 |
| Azithromycin vs. Nalidixic acid | 250.2657 | ＜0.0001 | Azithromycin vs. Nalidixic acid | 16.0000 | 0.0001 |
| Azithromycin vs. Ciprofloxacin | 267.1930 | ＜0.0001 | Azithromycin vs. Ciprofloxacin | 21.2073 | ＜0.0001 |
| Azithromycin vs. Gentamicin | 0.0000 | 1.0000 | Azithromycin vs. Gentamicin | 0.2229 | 0.8136 |
| Azithromycin vs. Streptomycin | 1.2269 | 0.3425 | Azithromycin vs. Streptomycin | 5.7143 | 0.0307 |
| Azithromycin vs. Chloramphenicol | 0.0914 | 0.8800 | Azithromycin vs. Chloramphenicol | 20.5714 | ＜0.0001 |
| Azithromycin vs. Florfenicol | 26.5351 | ＜0.0001 | Azithromycin vs. Florfenicol | 6.5455 | 0.0202 |
| Azithromycin vs. Tetracycline | 260.3098 | ＜0.0001 | Azithromycin vs. Tetracycline | 21.2073 | ＜0.0001 |
| Azithromycin vs. Telithromycin | 0.0914 | 0.8800 | Azithromycin vs. Telithromycin | 2.7413 | 0.1554 |
| Azithromycin vs. Clindamycin | 23.5508 | ＜0.0001 | Azithromycin vs. Clindamycin | 0.0000 | 1.0000 |
| Nalidixic acid vs. Ciprofloxacin | 0.9930 | 0.4259 | Nalidixic acid vs. Ciprofloxacin | 0.7273 | 0.6737 |
| Nalidixic acid vs. Gentamicin | 250.2657 | ＜0.0001 | Nalidixic acid vs. Gentamicin | 12.8291 | 0.0007 |
| Nalidixic acid vs. Streptomycin | 272.3113 | ＜0.0001 | Nalidixic acid vs. Streptomycin | 3.1921 | 0.1348 |
| Nalidixic acid vs. Chloramphenicol | 256.4103 | ＜0.0001 | Nalidixic acid vs. Chloramphenicol | 57.6000 | ＜0.0001 |
| Nalidixic acid vs. Florfenicol | 140.1791 | ＜0.0001 | Nalidixic acid vs. Florfenicol | 37.6718 | ＜0.0001 |
| Nalidixic acid vs. Tetracycline | 0.3346 | 0.7004 | Nalidixic acid vs. Tetracycline | 0.7273 | 0.6737 |
| Nalidixic acid vs. Telithromycin | 256.4103 | ＜0.0001 | Nalidixic acid vs. Telithromycin | 6.2374 | 0.0246 |
| Nalidixic acid vs. Clindamycin | 146.4103 | ＜0.0001 | Nalidixic acid vs. Clindamycin | 16.0000 | 0.0001 |
| Ciprofloxacin vs. Gentamicin | 267.1930 | ＜0.0001 | Ciprofloxacin vs. Gentamicin | 17.7231 | ＜0.0001 |
| Ciprofloxacin vs. Streptomycin | 289.4631 | ＜0.0001 | Ciprofloxacin vs. Streptomycin | 6.4000 | 0.0238 |
| Ciprofloxacin vs. Chloramphenicol | 273.4051 | ＜0.0001 | Ciprofloxacin vs. Chloramphenicol | 64.4211 | ＜0.0001 |
| Ciprofloxacin vs. Florfenicol | 155.1083 | ＜0.0001 | Ciprofloxacin vs. Florfenicol | 44.1000 | ＜0.0001 |
| Ciprofloxacin vs. Tetracycline | 0.1773 | 0.8338 | Ciprofloxacin vs. Tetracycline | 0.0000 | 1.0000 |
| Ciprofloxacin vs. Telithromycin | 273.4051 | ＜0.0001 | Ciprofloxacin vs. Telithromycin | 10.1895 | 0.0028 |
| Ciprofloxacin vs. Clindamycin | 161.5017 | ＜0.0001 | Ciprofloxacin vs. Clindamycin | 21.2073 | ＜0.0001 |
| Gentamicin vs. Streptomycin | 1.2269 | 0.3425 | Gentamicin vs. Streptomycin | 3.7403 | 0.0898 |
| Gentamicin vs. Chloramphenicol | 0.0914 | 0.8780 | Gentamicin vs. Chloramphenicol | 24.0000 | ＜0.0001 |
| Gentamicin vs. Florfenicol | 26.5351 | ＜0.0001 | Gentamicin vs. Florfenicol | 9.0000 | 0.0054 |
| Gentamicin vs. Tetracycline | 260.3098 | ＜0.0001 | Gentamicin vs. Tetracycline | 17.7231 | ＜0.0001 |
| Gentamicin vs. Telithromycin | 0.0914 | 0.8780 | Gentamicin vs. Telithromycin | 1.4162 | 0.3412 |
| Gentamicin vs. Clindamycin | 23.5508 | ＜0.0001 | Gentamicin vs. Clindamycin | 0.2229 | 0.8136 |
| Streptomycin vs. Chloramphenicol | 0.6514 | 0.5189 | Streptomycin vs. Chloramphenicol | 40.6957 | ＜0.0001 |
| Streptomycin vs. Florfenicol | 37.5881 | ＜0.0001 | Streptomycin vs. Florfenicol | 22.5000 | ＜0.0001 |
| Streptomycin vs. Tetracycline | 282.4942 | ＜0.0001 | Streptomycin vs. Tetracycline | 6.4000 | 0.0238 |
| Streptomycin vs. Telithromycin | 0.6514 | 0.5189 | Streptomycin vs. Telithromycin | 0.5750 | 0.6138 |
| Streptomycin vs. Clindamycin | 34.1283 | ＜0.0001 | Streptomycin vs. Clindamycin | 5.7143 | 0.0307 |
| Chloramphenicol vs. Florfenicol | 29.4257 | ＜0.0001 | Chloramphenicol vs. Florfenicol | 6.5455 | 0.0249 |
| Chloramphenicol vs. Tetracycline | 266.4961 | ＜0.0001 | Chloramphenicol vs. Tetracycline | 64.4211 | ＜0.0001 |
| Chloramphenicol vs. Telithromycin | 0.0000 | 1.0000 | Chloramphenicol vs. Telithromycin | 33.7959 | ＜0.0001 |
| Chloramphenicol vs. Clindamycin | 26.3025 | ＜0.0001 | Chloramphenicol vs. Clindamycin | 20.5714 | ＜0.0001 |
| Florfenicol vs. Tetracycline | 148.9919 | ＜0.0001 | Florfenicol vs. Tetracycline | 44.1000 | ＜0.0001 |
| Florfenicol vs. Telithromycin | 29.4257 | ＜0.0001 | Florfenicol vs. Telithromycin | 16.6864 | 0.0001 |
| Florfenicol vs. Clindamycin | 0.0999 | 0.8331 | Florfenicol vs. Clindamycin | 6.5455 | 0.0202 |
| Tetracycline vs. Telithromycin | 266.4962 | ＜0.0001 | Tetracycline vs. Telithromycin | 10.1895 | 0.0028 |
| Tetracycline vs. Clindamycin | 155.3230 | ＜0.0001 | Tetracycline vs. Clindamycin | 21.2073 | ＜0.0001 |
| Telithromycin vs. Clindamycin | 26.3025 | ＜0.0001 | Telithromycin vs. Clindamycin | 2.7413 | 0.1554 |
