# Supplementary figures and images for: Multilocus Sequence Types and Antimicrobial Resistance of Campylobacter jejuni and C. coli Isolates of Human Patients From Beijing, China, 2017–2018
Source: Front Microbiol. 2020 Oct 19;11:554784. doi: 10.3389/fmicb.2020.554784 (PMC7604515; doi:10.3389/fmicb.2020.554784)

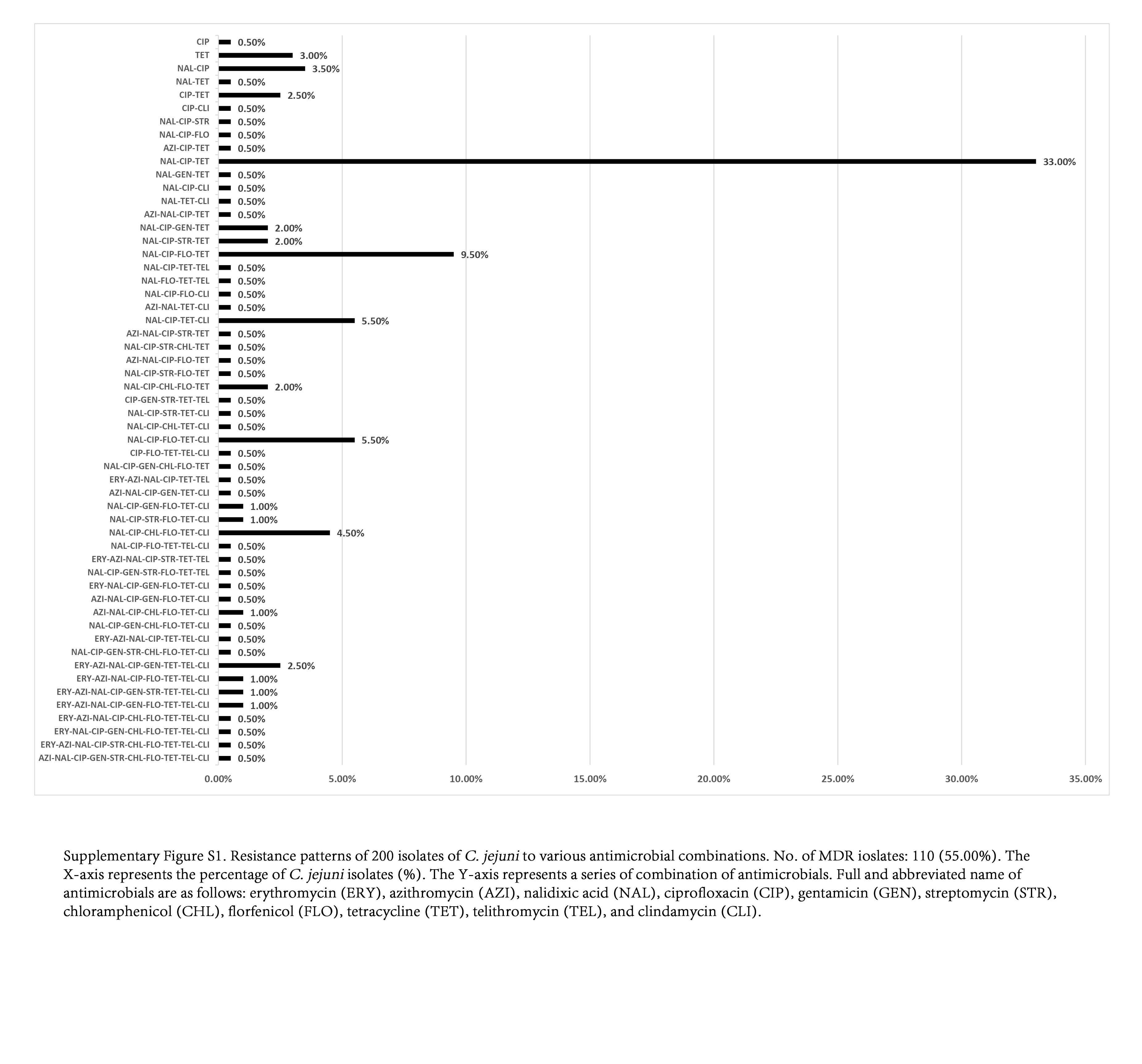

Supplement: Supplementary file 4 [file Image_1.TIF]

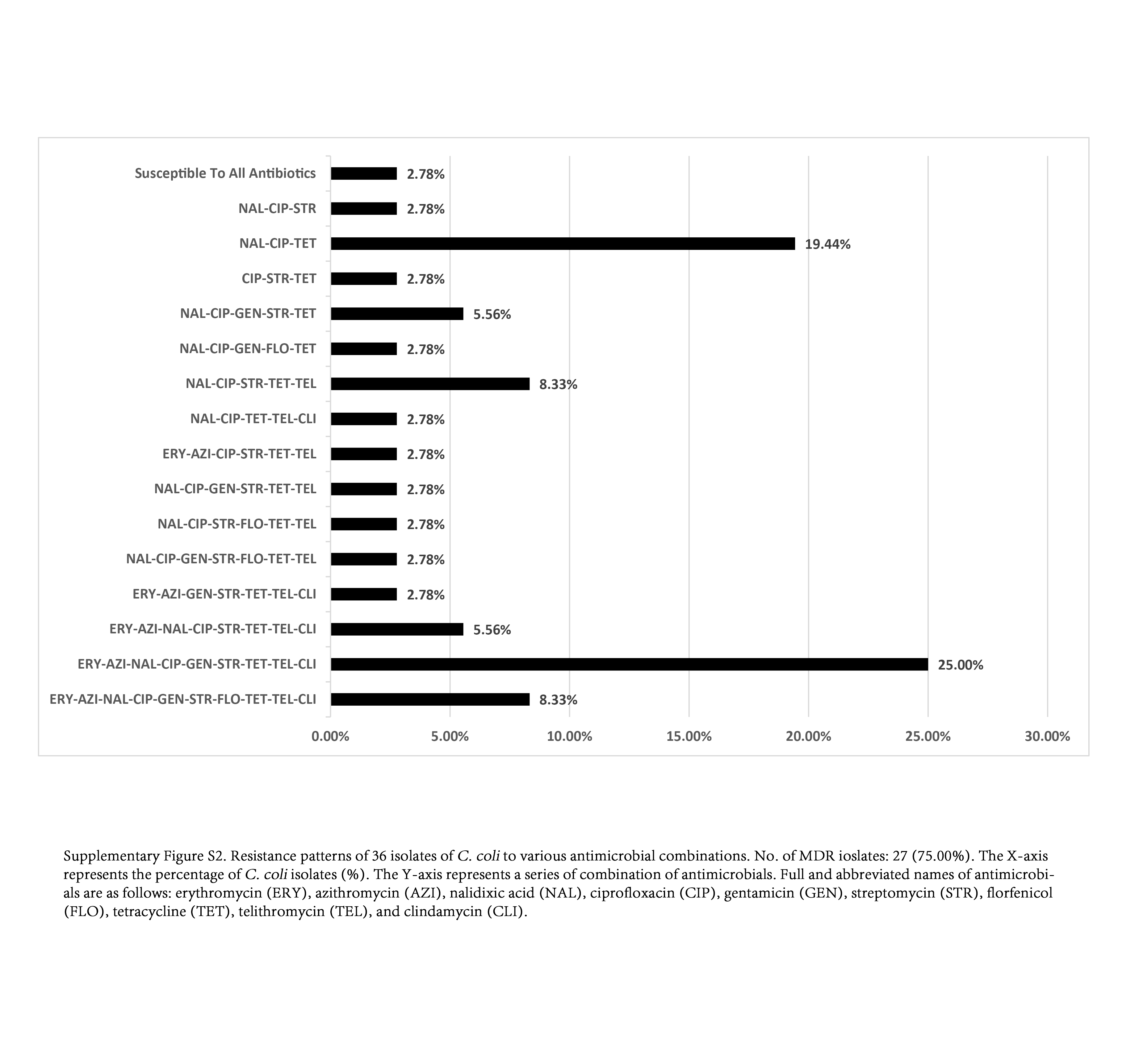

Supplement: Supplementary file 5 [file Image_2.TIF]
